# Urotensin II inhibited the proliferation of cardiac side population cells in mice during pressure overload by JNK-LRP6 signalling

**DOI:** 10.1111/jcmm.12230

**Published:** 2014-01-22

**Authors:** Zhidan Chen, Jiahong Xu, Yong Ye, Yang Li, Hui Gong, Guoping Zhang, Jian Wu, Jianguo jia, Ming Liu, Ying Chen, Chunjie Yang, Yu Tang, Yichun Zhu, Junbo Ge, Yunzeng Zou

**Affiliations:** a Shanghai Institute of Cardiovascular Diseases, Zhongshan Hospital and Institutes of Biomedical Sciences, Fudan UniversityShanghai, China; b Department of Cardiology, Tongji Hospital, Tongji University School of MedicineShanghai, China; c Department of Physiology and Pathophysiology, Shanghai Medical College, Fudan UniversityShanghai, China

**Keywords:** Urotensin II, CSPs, proliferation, JNK, LRP6

## Abstract

Cardiac side population cells (CSPs) are promising cell resource for the regeneration in diseased heart as intrinsic cardiac stem cells. However, the relative low ratio of CSPs in the heart limited the ability of CSPs to repair heart and improve cardiac function effectively under pathophysiological condition. Which factors limiting the proliferation of CSPs in diseased heart are unclear. Here, we show that urotensin II (UII) regulates the proliferation of CSPs by c-Jun *N*-terminal kinase (JNK) and low density lipoprotein receptor-related protein 6 (LRP6) signalling during pressure overload. Pressure overload greatly upregulated UII level in plasma, UII receptor (UT) antagonist, urantide, promoted CSPs proliferation and improved cardiac dysfunction during chronic pressure overload. In cultured CSPs subjected to mechanical stretch (MS), UII significantly inhibited the proliferation by UT. Nanofluidic proteomic immunoassay showed that it is the JNK activation, but not the extracellular signal-regulated kinase signalling, that involved in the UII-inhibited- proliferation of CSPs during pressure overload. Further analysis *in vitro* indicated UII-induced-phospho-JNK regulates phosphorylation of LRP6 in cultured CSPs after MS, which is important in the inhibitory effect of UII on the CSPs during pressure overload. In conclusion, UII inhibited the proliferation of CSPs by JNK/LRP6 signalling during pressure overload. Pharmacological inhibition of UII promotes CSPs proliferation in mice, offering a possible therapeutic approach for cardiac failure induced by pressure overload.

## Introduction

Heart failure remains a leading cause of morbidity and mortality, which is usually secondary to many cardiovascular diseases such as hypertension, myocardial infarction or atherosclerosis. The adult heart has long been thought to be a terminally differentiated organ without regenerative capacity [1]. Therefore, cardiac injury causes permanent myocardial loss and results in cardiac dysfunction [2]. However, the growing evidence reveal that cardiac progenitor cells may, in fact, exist [3]. More recently, cells expressing the stem cell markers, Sca1, c-kit or islet-1 in adult heart, have been identified to be capable of differentiation into cardiomyocytes [3–7]. Side population (SP) cells, one of the candidates for somatic stem cells, which are characterized by their intrinsic capacity to efflux Hoechst dye, were isolated from bone marrow (BM) [8] and other adult tissues including heart [9,10]. A recent study has shown that cardiac SP cells (CSPs) were able to differentiate into cardiomyocytes, endothelial cells, or smooth muscle cells *in vitro* and *in vivo*. Transplanted CSPs intravenously were observed to migrate and home in injured heart [11,12]. These evidence reveal that CSPs are promising cell resource for the regeneration in diseased heart as intrinsic cardiac stem cells. But the proportion of CSPs is very low at 1–2% in normal adult heart [12]. Although it increased more than threefold within acute myocardial injury [9,10], the relative low ratio of CSPs, to a great extent, limited the ability of CSPs to repair heart and improve cardiac function effectively under pathophysiological condition. The cellular mechanisms for regulating CSPs proliferation especially in diseased heart are unclear.

Some growth factors such as angiotensin II (Ang II) or urotension II (UII) have been reported to increase with the progression of myocardial remodelling response to pressure overload or ischaemic stimulus. Whether these factors exert anti-proliferation effect of CSPs which contributes to the limited proliferation of CSPs in diseased heart? To answer the question will help to develop novel strategy for promoting the proliferation of CSPs to effectively repair the diseased heart. Our recent study showed that UII inhibited the proliferation of CSPs through c-Jun *N*-terminal kinase (JNK) phosphorylation [13]. Human UII is an 11-amino-acid cyclic peptide that activates a G-protein coupled receptor GPR14 (Gq coupled receptor) named UII receptor (UT) [14]. Many studies have described a wide range of actions for human UII within the cardiovascular system, including cardiac hypertrophy [15], collagen synthesis [16] and positive inotropic effects [17]. Plasma UII level has been reported to be upregulated in patients with hypertension or congestive heart failure (CHF) [18,19]. Whether and how the high level UII inhibits the proliferation of CSPs response to cardiac injury are unclear.

Here, we build chronic pressure overload model to examine the UII level, observe the proliferation of CSPs and explore the potential mechanisms. This study will be helpful to find novel mechanisms for CSPs regulation and cardiac regeneration during pressure overload.

## Materials and methods

### Animal and surgical procedures

Pressure overload was produced by the transverse aorta constriction (TAC) as previously described [20]. C57BL/6 mice aged 8–10 weeks were subjected to TAC or sham operation after anaesthetized with ketamine (25 mg/kg IP). Briefly, after anaesthesia and artificial ventilation, the transverse aorta was constricted with the 7-0 nylon suture by ligating the aorta together with a blunted 27-gauge needle, which was pulled out later. From 2 to 4 weeks after TAC, urantide (30 μg/kg/day, Peptides International Inc., Louisville, KY, USA) or vehicle were respectively continuously administered by Alzet osmotic minipumps (Model 2002; DURECT, Cupertino, CA, USA) implanted subcutaneously into the back of mice. SP600125 or Dimethyl sulfoxide (DMSO) were injected intraperitoneally either with 40 mg/kg bodyweight every 3 days for 2 weeks. At 4 week after TAC, the number of CSPs was determined. All animal experimental protocols were approved by the Animal Care and Use Committee of Fudan University and in compliance with ‘Guidelines for the Care and Use of Laboratory Animals’ published by the National Academy Press (NIH Publication No. 85–23, revised 1996).

### ELISA

Blood sampling from mice was centrifuged within 30 min. at −4°C, and the plasma was stored at −80°C until assay. Plasma UII was determined by a specific, commercially available, high-sensitivity enzyme immunoassay with mouse EIA kit (Cat. no. EK-071-08; Phoenix Pharmaceuticals, Belmont, CA, USA). The antibody used in this assay doesn*t cross-react with endothelin-1 (ET-1), angiotensin II, adrenomedullin, calatonin gene-related peptide, brain natriuretic peptide, and bradykinin, and the cross-reaction with UII related peptide (URP) was <8%.

### Isolation of CSPs by fluorescence-activated cell sorting analysis

Cardiac side population cells were isolated and purified as previous study [13]. Briefly, cardiac cells isolated from C57BL/6 mice or neonatal rat were re-suspended at the density of 1.0 × 10^6^ cells/ml in PBS with 3% fetal calf serum (FBS). The cells were incubated in 1 μg/ml Hoechst 33342 dye for 60 min. at 37°C in the dark. After the incubation, cells were analysed for Hoechst 33342 dye efflux by flow cytometric analysis (EPICS ALTRA; Beckman Coulter, Indianapolis, IN, USA). Before analysis, 2 μg/ml of propidium iodide was added to distinguish live cells from dead cells. Hoechst 33342 dye was excited at 350 nm using UV laser. Fluorescent emission was detected through 450-nm BP (Hoechst blue) and 675-nm LP (Hoechst red) filters, respectively. Propidium iodide in cells was excited at 488 nm, and fluorescence emission was detected through a 620-nm BP filter. After sorted by flow cytometry, CSPs were frozen at −80°C or cultured on gelatin-coated dishes with Iscove*s Modified Dulbecco*s Medium (IMDM) supplemented with 10% FBS for the following experiments.

### Culture and mechanical stretch of CSPs

Cardiac side population cells were cultured on gelatin-coated dishes with IMDM supplemented with 10% FBS and then incubated in serum and antibiotic-free conditions in silicon-based plates pre-coated with collagen for 24 hrs before mechanical stretch (MS) to 120% for 24 hrs [20]. Urantide (10^−6^ mol/l), SP600125 or the same volume of PBS were administered in the medium and 30 min. later, the cells were subjected to MS and incubated with human UII (10^−7^ mol/l, cat. no. U7257; Sigma-Aldrich, St. Louis, MO, USA) for 6 hrs. The CSPs were collected for the following experiments.

### Constraction of UTR-cell line and Western Blot analysis

AD293 cells were stably transfected with human UTR/GPR14 pcDNA3.1+ (Missouri S&T cDNA Resource Center, Rolla, MO, USA) using FUGEN6 (Roche, Diagnostics Operations Inc., Indianapolis, IN, USA). Positive cells were selected by G418 (Sigma-Aldrich). UTR overexpression was identified by western blot with anti-GPR14 (Santa Cruz, Dallas, TX, USA). Lipoprotein receptor related protein 6 (LRP6) -pCS2 (Addgene, from Dr. Xi He) was transfected to UT cells to induce LRP6 overexperssion. For western blot analysis, isolated proteins were size-fractionated by SDS–PAGE and transferred to polyvinylidene difluoride membrane. And then the membranes were incubated with primary antibodies followed by incubation with a Horseradish Peroxidase (HRP)-conjugated secondary antibody (Jackson, West Grove, PA, USA). All antibodies used here were: phosphor-JNK, JNK and phosphor-LRP6 (1490), LRP6 (Cell Signalling, Danvers, MA, USA), GAPDH (Kangchen, Shanghai, China).

### Proliferation analysis

The proliferation of cultured CSPs was assessed by the CellTiter-Glo® Luminescent Cell Viability Assay (Promega, San Luis Obispo, CA, USA). The absorbance value of each well was determined at 490 nm by a microplate luminometer (Berthold Detection System, Orion II).

### Immunofluorescence and confocal image

Cells were fixed with 4% paraformaldehyde. After washing and blocking with PBS containing 2% bovine serum albumin (Sigma-Aldrich), cells were incubated with anti-phospho-LRP6 (Ser 1490) (1:100, Bioss Inc., Woburn, MA, USA) in 4°C overnight. They were washed in PBS and then incubated with FITC-conjugated anti-rabbit secondary antibody (1:1000; Invitrogen, Carlsbad, CA, USA). After washing in PBS, the cells were incubated with 4′,6-diamidino-2-phenylindole (10 μl/ml; Sigma-Aldrich) and observed under a confocal microscope (Zeiss, Munich, Germany).

### Nanofluidic proteomic immunoassay

We performed nanofluidic proteomic immunoassay (NIA) experiments with a firefly instrument (Protein Simple, Santa Clara, CA, USA) as previous study [21]. The cell lysates were clarified by centrifugation, and protein concentration was determined. Briefly, for each capillary analysis, we diluted the lysate sample to 0.05 mg/ml in Bicine/CHAPS Lysis Buffer and Sample Diluent (cat. no. 040-764; Protein Simple) with phosphatase inhibitor (DMSO Inhibitor mix, cat. no. 040-510; Protein Simple). We added 200 nl sample mix containing internal pI standards and calculated predicted pIs with Scansite 40. We ran each sample on a panel of different pH gradients (pH 3–10 and pH 2–11) to optimize the resolution of different peak patterns. The separated proteins were cross-linked to the capillary wall using UV light followed by immunoprobing with anti-ERK1/2 (Cell Signalling), anti-phospho-JNK (Cell Signalling) and anti-phosoho-LRP6 (1490; Cell Signalling). Nanofluidic proteomic immunoassay has previously been shown to be able to discriminate and quantify phosphorylated and unphosphorylated isoforms of extracellular signal-regulated kinas (ERK) in a single sample with a total ERK antibody. The areas under different peaks within a single tracing represented various ERK isoforms. To calculate the percentage of monophosphorylated ERK2 (identified here as a peak), we divided the area under the peak by the total area under all ERK peaks. HRP conjugated secondary antibodies were used to detect the signal. The digital image was analysed and peak area quantified with Compass software (Protein Simple).

### Statistical analysis

Data are expressed as means ± SEM. Multiple group comparison was performed by one-way anova followed by LSD procedure for comparison of means. Comparison between two groups under identical conditions was performed by the two-tailed Student*s *t*-test. A value of *P* < 0.05 was considered statistically significant.

## Results

### Plasma UII level is increased in pressure overload mice

**Fig. 1:**
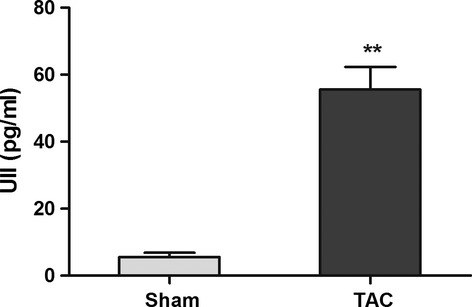
Plasma UII level is increased during pressure overload. Plasma UII level was examined with ELISA analysis. Values are expressed as mean ± SEM. Sham:*n* = 6. TAC:*n* = 6, ^*^^*^P < 0.05 *versus* sham mice.

Pressure overload was induced in C57BL/6 mice as reported previously for 4 weeks [5] by TAC. Haemodynamic parameters and myocardial function studies showed that TAC caused a significant increase in left ventricular systolic pressure in mice (Fig. S1A), when compared with sham mice, accompanied by reduced ejection fraction (EF; Fig. S1B). It revealed that the sustained pressure overload resulted in cardiac dysfunction. We then examined plasma UII level in sham and TAC mice by ELISA method. The result showed that pressure overload induced the significant increase in UII level in plasma (Fig. 1).We then examine CSPs number in TAC mice with or without UT receptor antagonist.

### UII inhibited the proliferation of CSPs by UT during mechanical stress *in vivo* or *in vitro*

**Fig. 2:**
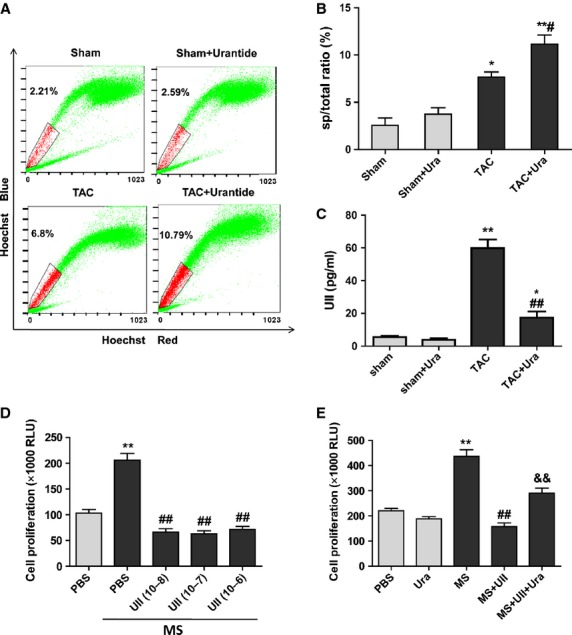
UII inhibits cardiac side population cells (CSPs) proliferation by UT during pressure overload. (**A**) The ratio of CSPs per mouse was analysed by fluorescence-activated cell sorting (FACS). Representative photographs are shown. (**B**) The ratio of CSPs per mouse was calculated by FACS. Urantide (30 μg/kg/day) or vehicle were, respectively, continuously administered by Alzet osmotic minipumps to mice from 2 to 4 weeks after transverse aorta constriction (TAC) or sham operation. (**C**) Plasma UII level was examined with ELISA analysis. Values are expressed as mean ± SEM. Sham:*n* = 9; Sham+Ura:*n* = 6; TAC:*n* = 6; TAC+Ura:*n* = 7. Ura: urantide. *P < 0.05, **P < 0.01 *versus* sham mice; ^#^*P* < 0.05, ^##^*P* < 0.01 *versus* TAC mice. (**D**) The proliferation of CSPs treated with different concentration of UII was determined during mechanical stretch (MS) by Cell Viability Assay. Cultured CSPs were treated with UII (0.01, 0.1, 1 μM) for 48 hrs after MS. (**E**) Urantide greatly reversed the anti-proliferation effect of UII on CSPs during MS. CSPs were pretreated with PBS or urantide (1 μM), UT antagonist, for 30 min., then subjected to MS and incubated with PBS or UII (0.1 μM) for 48 hrs. Values are expressed as mean ± SEM. *P < 0.05 *versus* control; ^#^*P* < 0.05 *versus* MS; ^&&^*P* < 0.05 *versus* MS plus UII. The experiment was repeated for three times.

Our previous study showed that UII inhibited the proliferation of CSPs *in vitro* and *in vivo* through UT receptor under physiological condition. Plasma UII level has been reported to be upregulated in patients with hypertension or CHF [18,19]. Here, we examined the proliferation of CSPs in pressure overload mice with or without urantide treatment. The results showed that pressure overload induced the increase in CSPs number by fluorescence-activated cell sorting (FACS) analysis, and urantide, UT antagonist, greatly promoted the proliferation of CSPs (Fig. 2A and B). Further analysis showed that UII antagonist, urantide sharply suppressed TAC-induced-upregulation of plasma UII level (Fig. 2C). It suggests that urantide not only inhibits the activation of UT but also downregulates UII level in plasma. Echocardiography analysis indicated that urantide significantly improved cardiac function characterized by increased EF after TAC (Fig. S2). To confirm whether high level UII inhibited the proliferation of CSPs after pressure overload, we detected the proliferation of CSPs with or without UII using *in vitro* device by which stretch stimuli can be imposed on cultured CSPs. The data revealed that MS induced the proliferation of CSPs, but UII greatly inhibited the effect from 0.01 to 1 μΜ (Fig. 2D), and urantide partly abolished the inhibitory effect of UII (0.1 μm; Fig. 2E). These data suggest UII inhibited the proliferation of CSPs by UT during pressure overload, and UT antagonist partly abolished the effect which may contribute to the cardiac protection.

### UT antagonist doesn*t affect the phosphorylation of ERK in CSPs during pressure overload

**Fig. 3:**
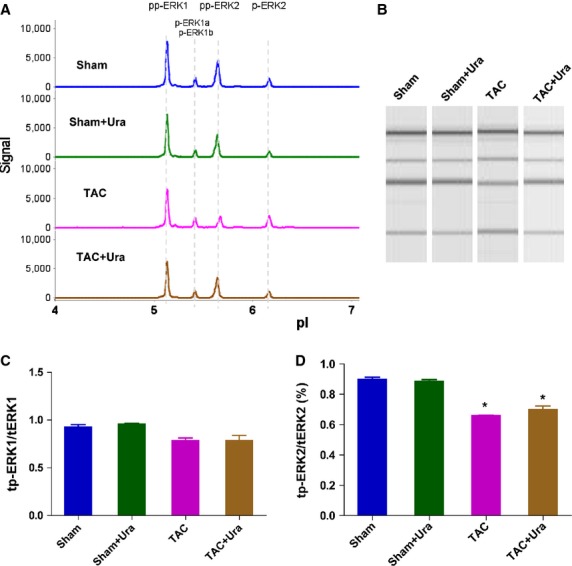
Urantide doesn*t affect the phosphorylation of extracellular signal-regulated kinas (ERK) in cardiac side population cells (CSPs) in pressure overload mice. (**A**) Multiple isoforms of Phospho-ERK was detected in CSPs by nanofluidic proteomic immunoassay (NIA). Peaks on the traces that represent phosphorylated isoforms are indicated. (**B**) NIA pseudoblot representation of ERK. (**C**) Bar graph of tp-ERK1/tERK1 by NIA quantification. tp-ERK1: total phosphorylation of ERK1. tERK1: total ERK1. (**D**) Bar graph of tp-ERK2/ERK2 by NIA quantification. tp-ERK2: total phosphorylation of ERK2. tERK2: total ERK2. Urantide (30 μg/kg/day) or vehicle were respectively continuously administered by Alzet osmotic minipumps to mice from 2 to 4 weeks after transverse aorta constriction (TAC) or sham operation, then CSPs were isolated from heart by fluorescence-activated cell sorting for NIA with antibody to phosphos-ERK. Values are expressed as mean ± SEM. Sham:*n* = 9. Sham+Ura:*n* = 6. TAC:*n* = 6. TAC+Ura:*n* = 7. Ura:urantide. *P < 0.05 *versus* sham mice.

Extracellular signal-regulated kinas pathway has been reported to be involved in the proliferation of various cells [22]. Our previous study showed that UII did not affect the activation of ERK1/2 in CSPs *in vitro*. We here confirm the effect in pressure overload mice. By FACS method, 10^4^–10^5^ CSPs per mouse could be isolated, which are not enough for western blot analysis. Therefore, NIA was used to detect ERK and distinct patterns of phosphoisomers in the CSPs from sham and TAC model. Nanofluidic proteomic immunoassay has been developed to quantify low-abundance protein isoforms in nanoliter volumes. A key feature of NIA is that it can be used to identify phosphorylated isoforms of a protein [21,23]. The results revealed that pressure overload did not affect the ratio of total phosphorylation of ERK1 to total ERK1 (Fig. 3A–C, Fig. S3), but it downregulated the ratio of total phosphorylation of ERK2 to total ERK2 (Fig. 3A, B and D, Fig. S3), However, the effects were not affected by urantide treatment. These data suggest that UT antagonist increased the amount of CSPs in pressure overload mice not by ERK pathway.

### UII promotes the activation of JNK in CSPs by UT during pressure overload *in vivo* and *in vitro*

**Fig. 4:**
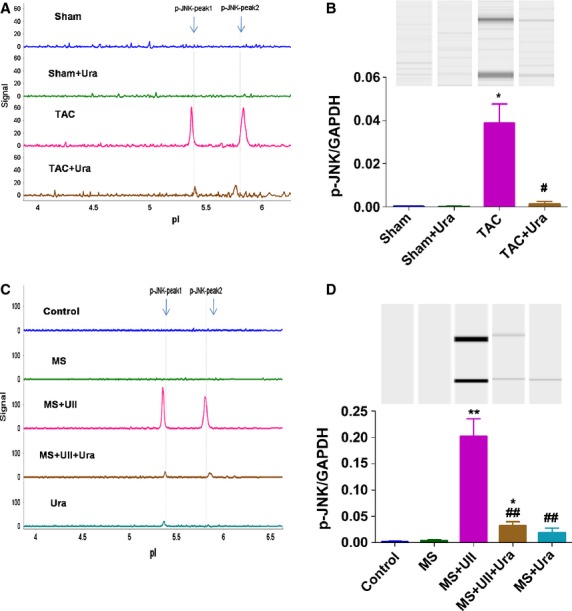
UII induces the activation of c-Jun *N*-terminal kinase (JNK) in cardiac side population cells (CSPs) during pressure overload. (**A**) Phospho-JNK was detected in CSPs isolated from transverse aorta constriction (TAC) or Sham mice by nanofluidic proteomic immunoassay (NIA). (**B**) NIA pseudoblot representation of phospho-JNK and bar graph of p-JNK/GAPDH in isolated CSPs by NIA quantification. Urantide (30 μg/kg/day) or vehicle were, respectively, continuously administered by Alzet osmotic minipumps to mice from 2 to 4 weeks after TAC or sham operation, then CSPs were isolated from heart by fluorescence-activated cell sorting for NIA with antibody to phosphos-JNK. Values are expressed as mean ± SEM. Sham:*n* = 9; Sham+Ura:*n* = 6; TAC:*n* = 6; TAC+Ura:*n* = 7. Ura: urantide. *P < 0.05 *versus* sham mice; ^#^*P* < 0.05 *versus* TAC mice. (**C**) Phospho-JNK was detected in cultured CSPs. (**D**) NIA pseudoblot representation of phospho-JNK and bar graph of p-JNK/GAPDH in cultured CSPs by NIA quantification. CSPs were pretreated with PBS or urantide (1 μM), for 30 min., then subjected to mechanical stretch (MS) and incubated with or without UII (0.1 μM) for 30 min. Peaks on the traces that represent phosphorylated isoforms are indicated. Values are expressed as mean ± SEM. *P < 0.05, **P < 0.01 *versus* MS; ^#^*P* < 0.05, ^##^*P* < 0.01 *versus* MS plus UII. The experiment was repeated for at least three times.

Our recent study showed that UII inhibited the proliferation of CSPs *in vitro* by activation of JNK [13]. We next examined phosphoisomers of JNK in CSPs in sham or TAC mice by NIA. The results revealed that pressure overload dramatically promoted the activation of JNK in CSPs, but UT antagonist, urantide, greatly inhibited the effects (Fig. 4A and B, Fig. S4A). To explore whether the elevated UII level during pressure overload induces the activation of JNK in CSPs, we cultured CSPs subjected to MS and detected the phosphorylation of JNK. Mechanical stretch alone induced little increase in phosphorylation of JNK, but UII sharply increased the phosphorylation of JNK in CSPs during MS, while urantide significantly inhibited the effect (Fig. 4C and D, Fig. S4B). These data suggest that UII inhibits the proliferation of CSPs during pressure overload mice by UT/JNK pathway.

### UII inhibits the phosphorylation of LRP6 induced by MS in CSPs

**Fig. 5:**
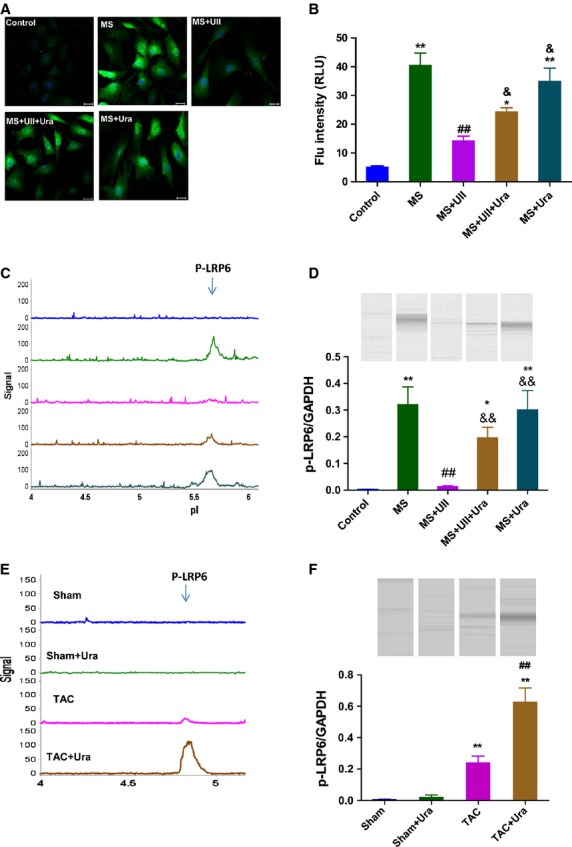
UII inhibits the phosphorylation of LRP6 in cardiac side population cells (CSPs) by UT/JNK signaling during mechanical stretch. (**A**) Immunofluorescent images revealed that positive signal (green) could be observed in cultured CSPs with antibody to p-LRP6 (1:200); bar: 20 μm. (**B**) Quantitative evaluation of p-LRP6 by analysis of fluorescence intensity. (**C**) P-LRP6 was detected in cultured CSPs by nanofluidic proteomic immunoassay (NIA). Peaks on the traces that represent phosphorylated isoforms of LRP6 are indicated. (**D**) NIA pseudoblot representation of p-LRP6 and bar graph of p-LRP6/GAPDH in cultured CSPs by NIA quantification. Cultured CSPs were pre-treated with PBS, urantide (Ura, 1 μM) or SP600125 (SP6, 5 μM), respectively, for 30 min, then subjected to mechanical stretch (MS) and incubated with PBS or UII (0.1 μM) for 3 hrs. Values are expressed as mean ± SEM. **P < 0.01 *versus* control; ^##^*P* < 0.01 *versus* control; ^#^*P* < 0.05 *versus* MS; ^&^*P* < 0.05 *versus* MS plus UII; ^&&^*P* < 0.01 *versus* MS plus UII. The experiment was repeated for at least three times. (**E**) P-LRP6 was detected in CSPs isolated from transverse aorta constriction (TAC) or Sham mice by NIA. (**F**) NIA pseudoblot representation of p-LRP6 and bar graph of p-LRP6/GAPDH in isolated CSPs by NIA quantification. Values are expressed as mean ± SEM. Sham:*n* = 9; Sham+Ura:*n* = 6; TAC:*n* = 6; TAC+Ura:*n* = 7. Ura: urantide. **P < 0.01 *versus* Sham mice; ^##^*P* < 0.01 *versus* TAC mice.

Lipoprotein receptor related protein 6 phosphorylation and subsequently induced-β-catenin signalling have been reported to be involved in the mitotic program of cells [24]. We next detect the phosphorylation of LRP6 in CSPs treated with UII during MS by immunofluorescence analysis. The results showed that MS greatly upregulated the phosphorylation of LRP6 level, but UII dramatically inhibited the effect. Urotensin II receptor antagonist, urantide, partly abolished the inhibitory effect of UII on the phosphorylation of LRP6 in CSPs after MS (Fig. 5A and B). We then confirm the results by NIA method. It is consistent with the result from immofluorescence analysis (Fig. 5C and D). Further research by NIA showed that urantide upregulated the p-LRP6 level in isolated CSPs from TAC model (Fig. 5E). It suggests that UII inhibits the phosphorylation of LRP6 induced by MS in CSPs, which may be related to its negative effect on the proliferation of CSPs.

### UII inhibited the phosphorylation of LRP6 by activating JNK signaling during MS

**Fig. 6:**
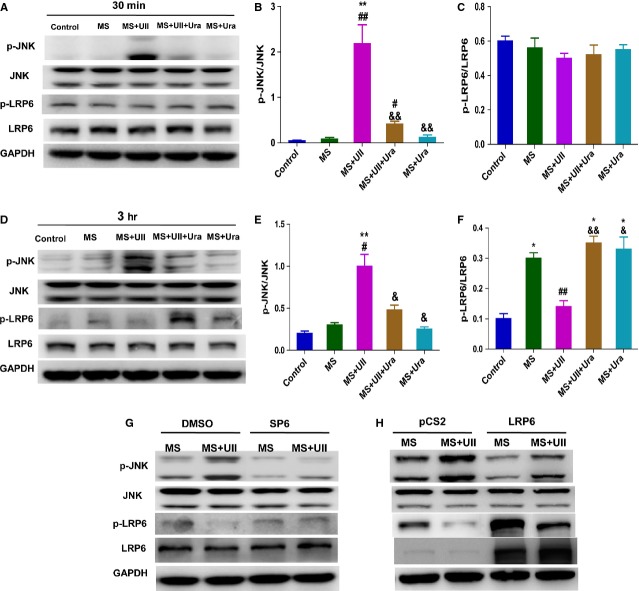
UII inhibits the phosphorylation of LRP6 in UT cell line by activating JNK during mechanical stretch. Western blot analysis for p-LRP6 and p-JNK levels. UT cells were pre-treated with Urantide (Ura, 1 μM) for 30 min., then subjected to MS and treated with UII for 30 min. and 3 hrs. (**A**) Representative picture of p-LRP6 and p-JNK level at 30 min. after UII treatment during MS. Quantitative evaluation of p-JNK (**B**) and p-LRP6 (**C**) by analysis of Western blot. (**D**) Representative picture of p-LRP6 and p-JNK level at 3 hrs after UII treatment during MS. Quantitative evaluation of p-JNK (**E**) and p-LRP6 (**F**) by analysis of Western blot. P-JNK and p-LRP6 were analysed in UT cells pre-treated with SP600125 (SP6, 5 μM; **G**) or overexpressed LRP6 (**H**) with or without UII (0.1 μM) during MS. UT cells were pre-treated with SP600125 or DMSO for 30 min., then subjected to MS and treated with UII for 3 hrs. UT cells were overexpressed with LRP6-pCS2 or pCS2 for 48 hrs, then subjected to MS and treated with UII for 3 hrs. Values are expressed as mean ± SEM. *P < 0.05, **P < 0.01 *versus* control. ^#^*P* < 0.05, ^##^*P* < 0.01 *versus* MS. ^&^*P* < 0.05, ^&&^*P* < 0.01 *versus* MS plus UII. The experiment was repeated for at least three times.

Directed phosphorylation of LRP6 by mitogen-activated protein kinases (MAPKs) such as ERK1/2 and JNK1 depends on cell type and the external stimulus [25]. To explore whether UII inhibits the phosphorylation of LRP6 during MS by activating JNK, we firstly observed p-LRP6 and p-JNK level at 30 min. and 3 hrs in UT cell line after UII treatment during MS with or without urantide pretreatment. The results showed that p-JNK was significantly upregulated at 30 min. and 3 hrs after UII treatment during MS, but p-LRP6 was only inhibited by UII at 3 hrs during MS. Urantide, respectively, partly abolished the effect (Fig. 6A–F). Further study showed that SP600125, JNK inhibitor, greatly reversed the p-LRP6 level after UII treatment during MS, but LRP6 overexpression did not affect the upregulation of p-JNK induced by UII during MS, although it slightly decreased p-JNK level after MS (Fig. 6G and H). The data suggest that UII inhibits the phosphorylation of LRP6 during MS by activating JNK.

### JNK inhibitor or LRP6 overexpression greatly improved the inhibitory effect of UII on the proliferation of CSPs during MS

**Fig. 7:**
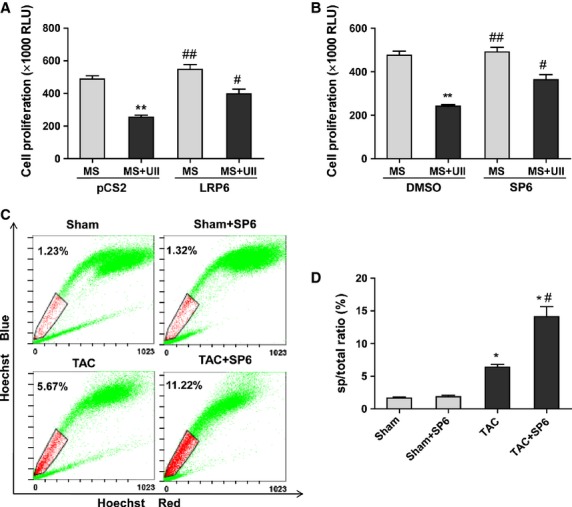
UII inhibits the proliferation of cardiac side population cells (CSPs) by c-Jun *N*-terminal kinase (JNK)-LRP6 signalling during mechanical stretch. CSPs proliferation was determined by Cell Viability Assay. Cultured CSPs were overexpressed LRP6 (**A**) or pretreated with SP600125 (SP6, 5 μM; **B**) with or without UII (0.1 μM) during mechanical stretch (MS). CSPs were pre-treated with SP600125 or DMSO for 30 min., then subjected to MS and treated with UII for 48 hrs. CSPs were overexpressed with LRP6-pCS2 or pCS2 for 48 hrs, then subjected to MS and treated with UII for 48 hrs. Values are expressed as mean ± SEM. **P < 0.01 *versus* MS. ^#^*P* < 0.05 *versus* MS plus UII. The experiment was repeated for at least three times. (**C**) The ratio of CSPs per mouse was analysed by fluorescence-activated cell sorting (FACS). Representative photographs are shown. (**D**) The ratio of CSPs per mouse was calculated by FACS. SP600125 (SP6) or DMSO were injected intraperitoneally either with 40 mg/kg bodyweight every 3 days for 2 weeks. At 4 weeks after transverse aorta constriction (TAC) or sham operation, the number of CSPs was determined. Values are expressed as mean ± SEM. Sham:*n* = 5; Sham+SP6:*n* = 6; TAC:*n* = 6; TAC+SP6:*n* = 6. Ura: urantide. *P < 0.05 *versus* sham mice; ^#^*P* < 0.05 *versus* TAC mice.

We then examined whether UII inhibited the proliferation of CSPs by JNK-LRP6 signalling after MS. The results showed that either SP600125 or LRP6 overexpression partly abolished the anti-proliferation effect of UII *in vitro* (Fig. 7A and B). Further analysis *in vivo* revealed that SP600125, a JNK inhibitor, greatly upregualted CSPs number in mice subjected to TAC (Fig. 7C and D). The analysis showed that JNK inhibitor attenuated the negative effect of UII on the proliferation of CSPs after MS. These data indicate that UII inhibited the proliferation of CSPs after MS by JNK-regulated-LRP6-signalling.

## Discussion

Our data indicated that plasma UII level was greatly increased in pressure overload mice. Urotensin II inhibited the proliferation of CSPs through UT signalling in this model. Further research revealed UII induced the phosphorylation of JNK and subsequently decreased phospho-LRP6 level, which contributed to the inhibitory effect of UII on the proliferation of CSPs in pressure overload mice.

After myocardial injury, irreversible cardiomyocytes loss may lead to the development of the progressive heart failure [26]. Cardiac side population cells represent a cardiac stem/progenitor cell population that can be enriched by the isolation of the Hoechst 33342 effluxing fraction [27]. As one kind of resident cardiac stem cells, CSPs can migrate and differentiate into functional cardiomyocytes to regenerate myocardium in response to the ischaemia-induced factors (JCB). It seems that CSPs are the promising candidate for repopulating the injured heart with new myocytes owing to their cardiomyogenic potential. The proportion of CSPs decreased rapidly during the seven postnatal days in mice [28], but it has been observed to increase more than threefold within 3 days of myocardial injury [10]. Following myocardial infarction in adult mice, resident CSP populations are acutely depleted in the injured as well as non-injured regions, which would subsequently return to baseline levels within 7 days [29]. The cellular mechanism underlying this cardiac progenitor cell pool fluctuation in diseased heart remains unknown. Our recent study revealed that UII greatly inhibited the proliferation of CSPs although it did not affect their differentiation under physiological condition [13]. Urotensin II is a somatostatin-like cyclic peptide synthesized by proteolytic cleavage from prepro-UII, and exists together with its receptor, GPR14, named UT in the heart [14]. Although the expression of UII and UT is relatively low in normal myocardium, UII and UT are highly expressed in cardiac tissue from patients with CHF. Plasma UII level has been reported to correlate positively with disease severity (New York Heart Association class) and inversely with cardiac function [19] in patients with hypertension or CHF [18,19]. Human UII also causes positive inotropy in human and rat myocardium [13,17], hypertrophic response in cardiamyocytes [15], mitogenesis in vascular smooth muscle cells [30] and cardiac fibroblasts [31]. However, few reports focus on the effect of UII on cardiac stem cell during cardiac injury.

In the present study, we built pressure overload model to induce cardiac remodelling in mice and examined the plasma UII level *in vivo*. At 4 weeks after TAC, EF was decreased to less than 50% in mice. It revealed that TAC induced cardiac dysfuntion in mice owing to the chronic pressure overload. Plasma UII was increased fivefold than that in sham group. CSPs were increased significantly in pressure overload mice in spite of higher level of UII. Interestingly, UT antagonist, urantide, further promoted the increase in CSPs number and improve cardiac function after pressure overload. Interestingly, we obseved that urantide greatly inhibited TAC-induced-upregulation of UII level in plasma. It suggests that urantide promotes CSPs proliferation during pressure overload not only by inhibiting the activation of UT but also by inhibiting the upregulation of UII. Some researchers thought that the existence of rodent UII is still controversial. Sugo *et al*. reported they failed to detect mature UII peptide but only identified URP by HPLC in rat brain tissue [32]. The mature forms of UII and URP share high homology although the precursor proteins of UII and URP are quite different and show only 18.8% amino acid homology. However, Dubessy *et al*. showed that the mature form of UII, comprising 17 residues, is produced in the mouse brain, by HPLC/RIA analysis [33]. It suggests that mature UII does exist in mouse tissues.

To explore whether UII inhibited the proliferation of CSPs by UT during pressure overload, we cultured CSPs with or without MS *in vitro* and detected the proliferation of CSPs. The results revealed that MS promoted the proliferation of CSPs, but UII inhibited the effect, while urantide partly abolished the inhibitory effect of UII on the proliferation of CSPs. It suggests that UII inhibits the proliferation of CSPs through UT signalling pathway during pressure overload. These results imply there exist other factors that promote the proliferation of CSPs under pressure overload, which overlay the inhibitory effect of UII.

To explore the potential mechanisms, we using NIA analysis to detect the phospho-ERK and phospho-JNK level *in vivo* [21]. Nanofluidic proteomic immunoassay is a highly sensitive and reproducible nanoscale technology to quantify low-abundance protein isoforms in nanoliter volumes, and it combines isoelectric protein focusing and antibody detection. A key feature of NIA is that it can be used to identify phosphorylated isoforms of a protein [21,23]. For the small population of CSPs, we used the analysis instead of western blot analysis to detecte the level of p-JNK and p-ERK. c-Jun *N*-terminal kinase and ERK are usually involved in the proliferation or apoptosis of various cells [34]. Our recent study showed it UII inhibited the proliferation of CSPs by JNK activation under physiological condition. The present study revealed that phospho-ERK level in CSPs was downregulated at 4 week after pressure overload, but UT antagonist, urantide didn*t affect it. However, urantide greatly inhibited the phosphorylation of JNK in CSPs induced by pressure overload. In cultured CSPs, UII dramatically upregulated the level of phospho-JNK induced by MS, and urantide partly abolished the effect. It suggests that it is the JNK activation, but not the ERK phosphorylation, that involved in the UII-inhibited-proliferation of CSPs during pressure overload.

Low density LRP6 is a coreceptor of WNTs and a key regulator of the WNT/β-catenin pathway. Upon phosphorylation of LRP6 within its intracellular PPPS/TP motifs, cytoplasmic β-catenin accumulates and translocates into the nucleus, where it binds to the N terminus of LEF/TCF (lymphoid enhancer factor/T cell factor) transcription factors and regulate TCF-target genes such as cMyc and Cyclin D1 to maintain progenitor cell proliferation [35]. Several candidate kinases such as G-protein-coupled receptor kinases GSK3 [36], GRK5 [37,38] and CK1γ have been identified to mediate phosphorylation of LRP6. Recent study reported that MAPKs, such as p38, ERK1/2 and JNK are sufficient and required for the direct phosphorylation of PPPS/TP motifs of LRP6 [25]. Interestingly, our study revealed that UII greatly inhibited the phosphorylation of LRP6 induced by MS through UT although it could further promote the activation of JNK in CSPs, but JNK inhibitor partly abolished the effect of UII in cultured CSPs under MS. Previous study showed that JNK inhibitor significantly decreased the phospho-LRP6 level induced by WNT 3a in HEK293 cells [25]. It suggests that regulation of JNK on the phosphorylation of LRP6 may be dependent on the extracellular stimulation or cell type. Our further study showed that JNK inhibitor or LRP6 overexpression significantly improve the anti-proliferation effect of UII on CSPs. The limitation of the present study is that we did not construct LRP6 overexpression mice or prepro-UII knockout mice subjected to TAC and study the proliferation of CSPs and cardiac function.

Therefore, in the present study, UII was increased greatly in plasma after pressure overload, and the high level of UII activated JNK. The phospho-JNK subsequently inhibited phosphorylation of LRP6, which resulted in the inhibitory effect of UII on the proliferation of CSPs after pressure overload. To some extent, it can explain why CSPs could not sharply proliferate and regenerate cardiomyocytes during pressure overload. These findings provide an understanding on the mechanisms responsible for the UII-regulated-proliferation of CSPs under pressure overload. Pharmacological inhibition of UII promotes CSPs proliferation in mice, offering a possible therapeutic approach for cardiac failure induced by pressure overload.

## Supporting information

**Figure S1** Pressure overload induces cardiac dysfunction.

**Figure S2** Urantide improves cardiac function during pressure overload.

**Figure S3** ERK was detected in isolated CSPs by NIA.

**Figure S4** GAPDH was detected in CSPs by NIA.
