# Supplementary figures and images for: Urotensin II inhibited the proliferation of cardiac side population cells in mice during pressure overload by JNK-LRP6 signalling
Source: J Cell Mol Med. 2014 Jan 22;18(5):852–62. doi: 10.1111/jcmm.12230 (PMC4119391; doi:10.1111/jcmm.12230)

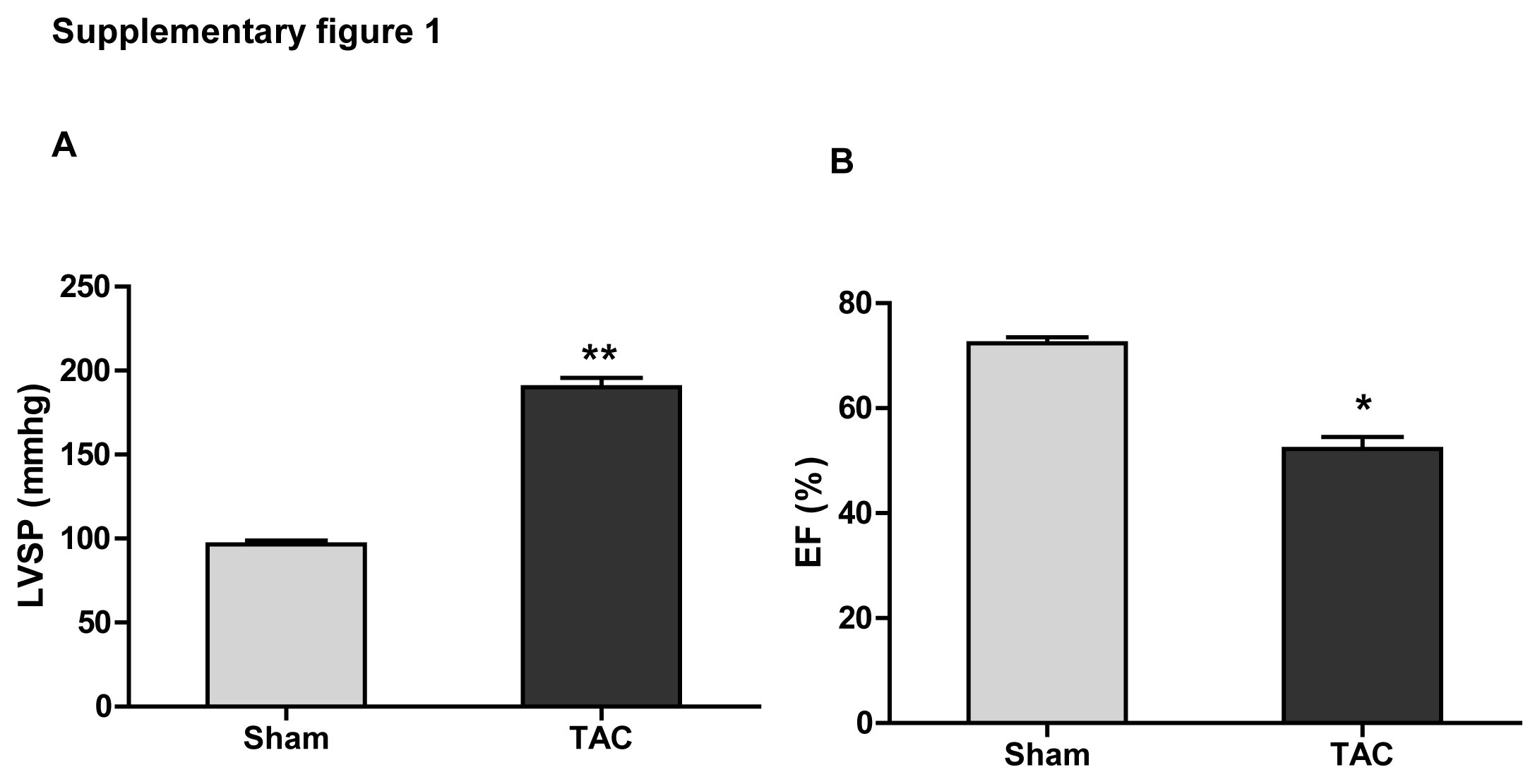

Supplement: Supplementary file 1 — Figure S1 Pressure overload induces cardiac dysfunction. [file jcmm0018-0852-SD1.tif]

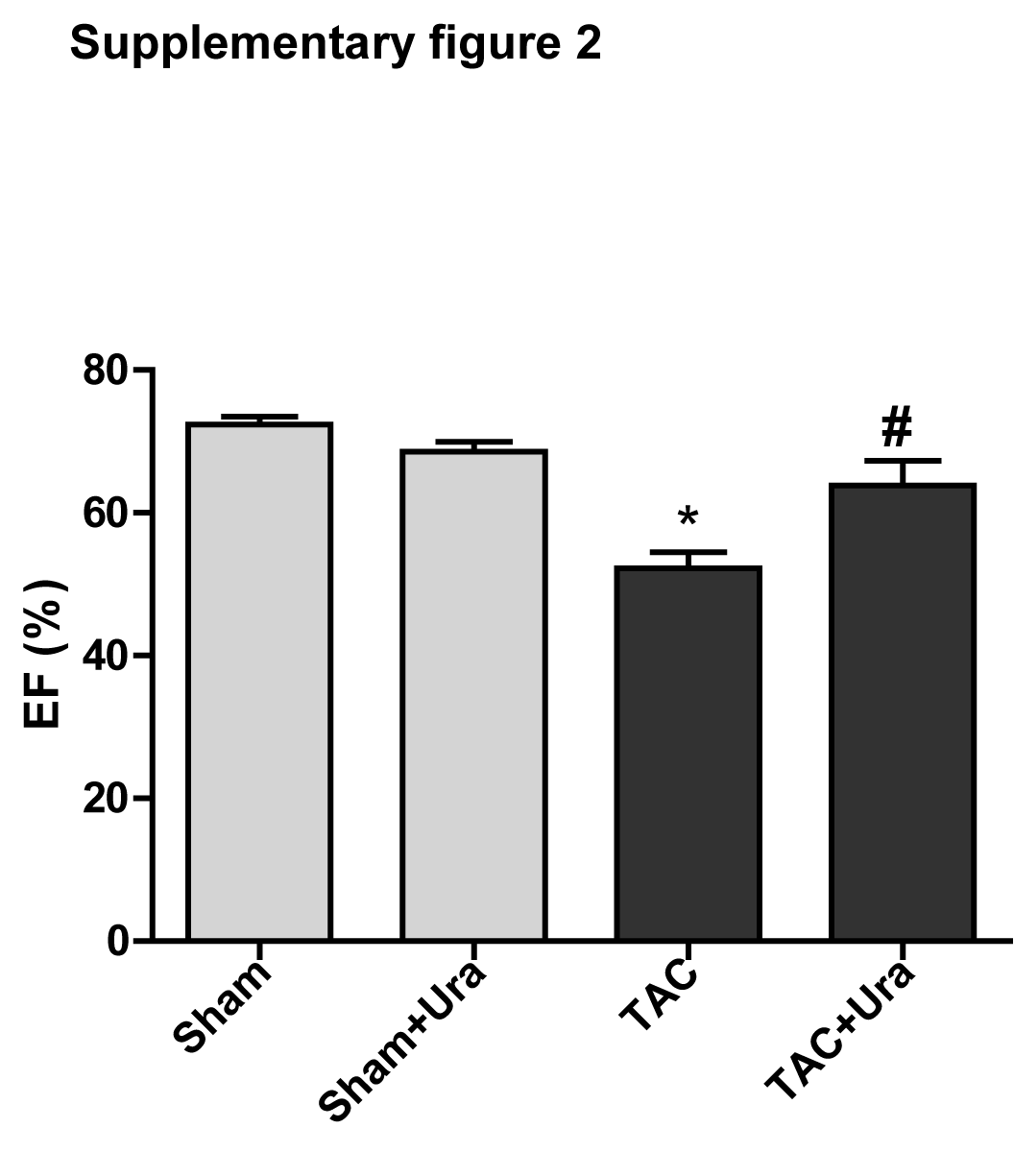

Supplement: Supplementary file 2 — Figure S2 Urantide improves cardiac function during pressure overload. [file jcmm0018-0852-SD2.tif]

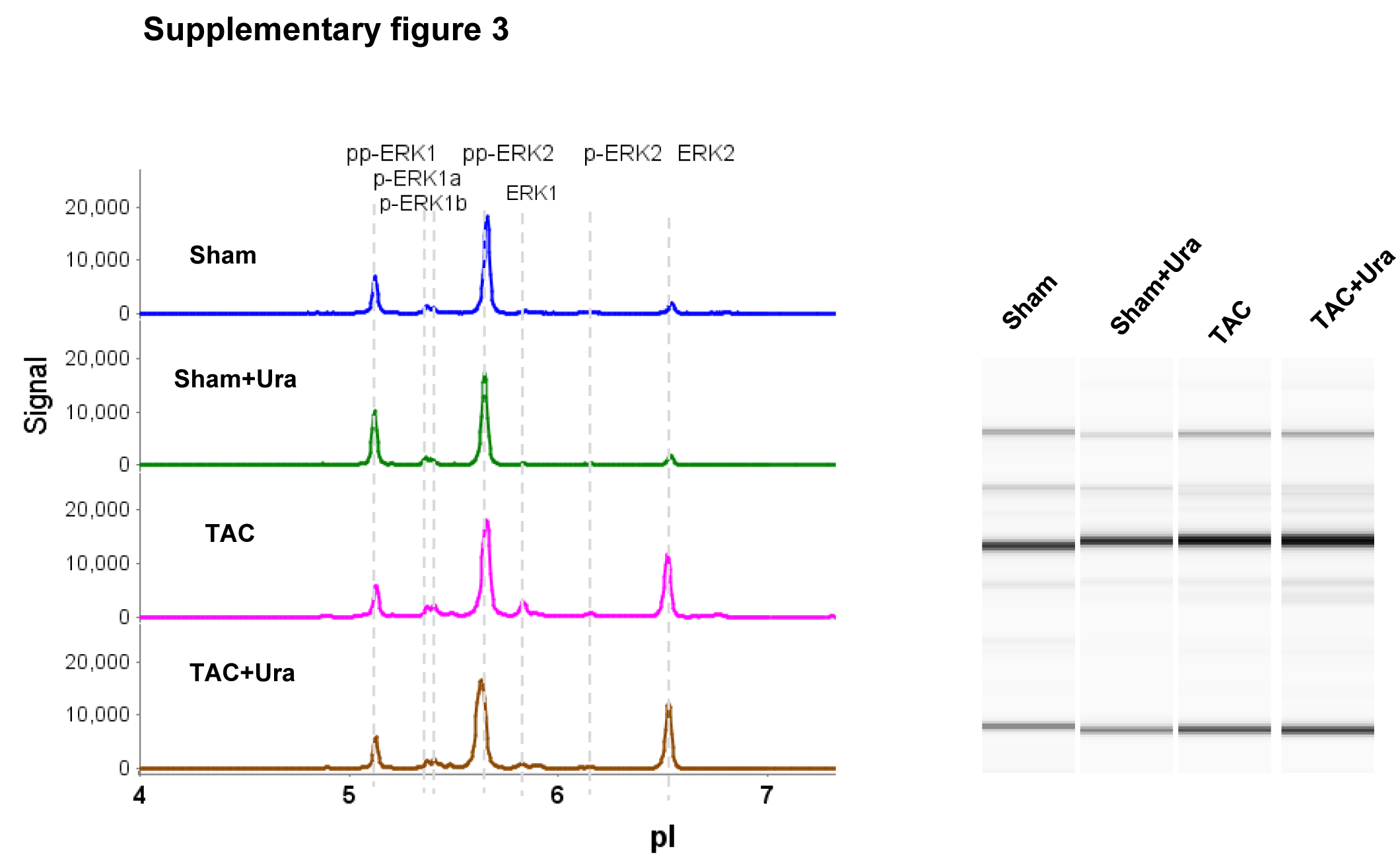

Supplement: Supplementary file 3 — Figure S3 ERK was detected in isolated CSPs by NIA. [file jcmm0018-0852-SD3.tif]

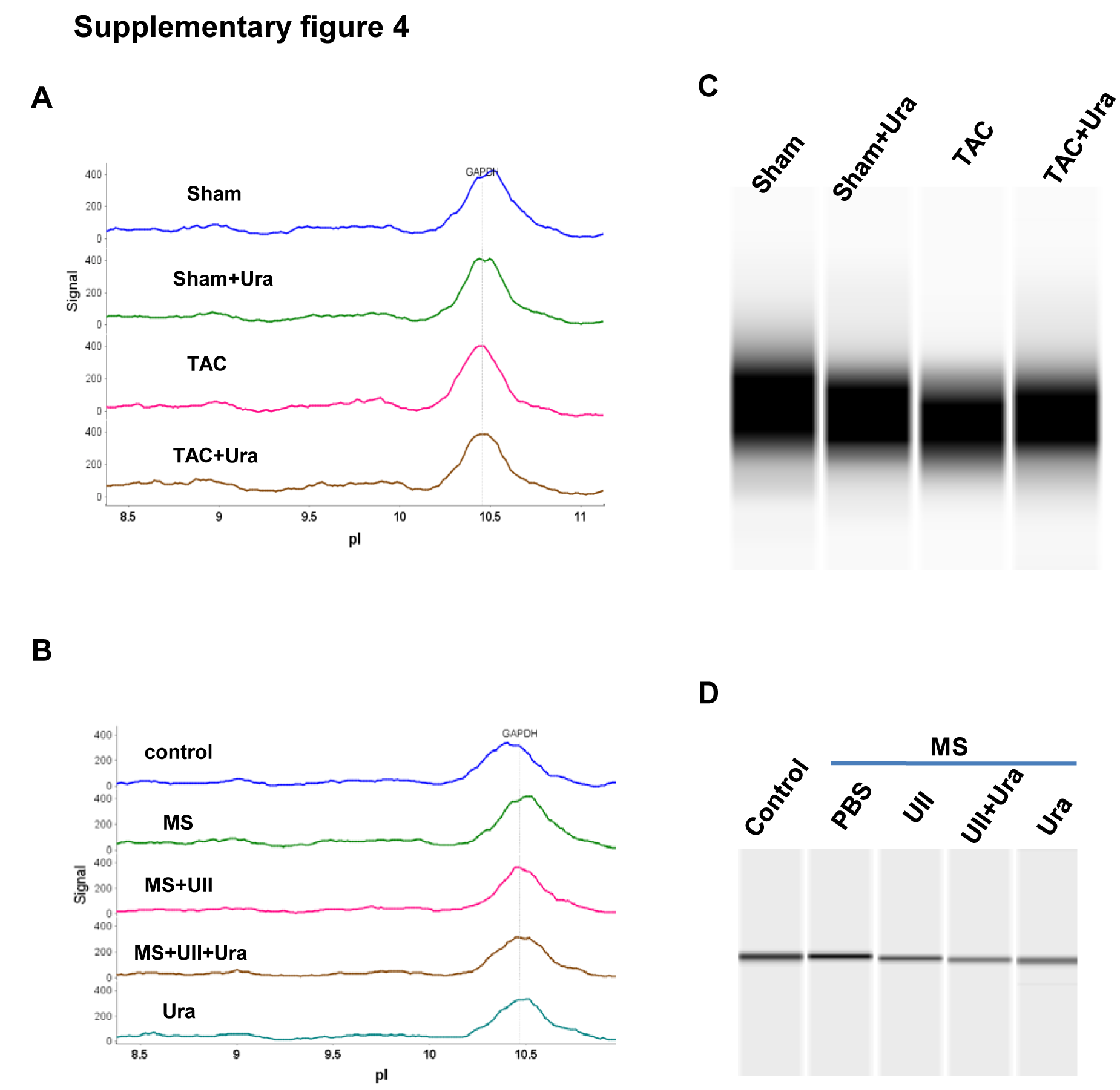

Supplement: Supplementary file 4 — Figure S4 GAPDH was detected in CSPs by NIA. [file jcmm0018-0852-SD4.tif]
