# Supplementary material for: Urotensin II inhibited the proliferation of cardiac side population cells in mice during pressure overload by JNK-LRP6 signalling
Source: J Cell Mol Med. 2014 Jan 22;18(5):852–62. doi: 10.1111/jcmm.12230 (PMC4119391; doi:10.1111/jcmm.12230)
Supplement: Supplementary file 5 — Figure S4 GAPDH was detected in CSPs by NIA. [file jcmm0018-0852-SD5.docx]

Supplementary materials

Supplementary methods

**Left ventricular systolic pressure and cardiac function assessment**

Echocardiography was performed to examine cardiac function of all the mice after TAC or sham for 4 weeks. After inhalation anesthesia of isoflurance, a parasternal long-axis view was obtained for M-mode imaging and ejection fraction (EF) was assessed with RMV 707 scanhead on the Vevo 770 (VisualSonics Inc, Toronto, Canada). Heart rate was maintained at more than 450 beats per min. Left ventricular systolic pressure (LVSP) was measured by a 1.4F pressure catheter (SPR 671, Millar Instruments) inserted into the aorta and LV through the right common carotid artery. The transducer was connected to Powerlab system (AD Instruments, Castle Hill, Australia) to record left ventricular systolic pressure (LVSP).

Supplementary figure legends

Supplementary figure 1. Pressure overload induces cardiac dysfunction.

(A) LVSP was measured by Powerlab system with a 1.4F pressure catheter inserted into the aorta and LV at 4 week after TAC. (B) EF was analyzed by ecocardiography. Pressure overload was produced by the transverse aorta constriction (TAC), mice subjected to sham operation were as control. Values are expressed as mean±S.E.M. Sham:n=6. TAC:n=6, ** p<0.01 vs sham mice;

Supplementary figure 2. Urantide improves cardiac function during pressure overload.

EF was analyzed by ecocardiography. Urantide (30μg/kg/day) or vehicle were respectively continuously administered by Alzet osmotic minipumps to mice from 2 weeks to 4 weeks after TAC or sham operation. Values are expressed as mean±S.E.M. Sham:n=9; Sham+Ura: n=6; TAC: n=6; TAC+Ura: n=7. Ura: urantide. * p<0.05 vs sham mice; ^#^ p<0.05 vs TAC mice.

Supplementary figure 3. ERK was detected in isolated CSPs by NIA.

1. Peaks on the traces that represent muiltiple isoforms of ERK are indicated. (B) NIA pseudoblot representation of ERK. Urantide (30μg/kg/day) or vehicle were respectively continuously administered by Alzet osmotic minipumps to mice from 2 weeks to 4 weeks after TAC or sham operation, then CSPs were isolated from heart by FACS for NIA with antibody to ERK. ERK1:non-phosphorylation of ERK1. ERK2:non-phosphorylation of ERK2.

Supplementary figure 4. GAPDH was detected in CSPs by NIA.

Peaks on the traces that represent GAPDH are indicated in isolated CSPs at 4 week after TAC or sham operation (A) or in cultured CSPs (B). NIA pseudoblot representation of GAPDH in isolated CSPs at 4 week after TAC or sham operation (C) or in cultured CSPs (D). Urantide (30μg/kg/day) or vehicle were respectively continuously administered by Alzet osmotic minipumps to mice from 2 weeks to 4 weeks after TAC or sham operation, then CSPs were isolated from heart by FACS for NIA with antibody to GAPDH. Cultured CSPs were pretreated with PBS, urantide (Ura, 1μM) for 30min, then subjected to mechanical stretch (MS) and incubated with or without UII (0.1μM) for 30min.
